# Contrast-enhanced ultrasound for non-invasive differential diagnosis of unclear left atrial mass

**DOI:** 10.1007/s10554-023-02926-7

**Published:** 2023-09-04

**Authors:** Martin Christa, Jonas Müntze, Björn Lengenfelder, Peter Nordbeck

**Affiliations:** Department of Internal Medicine I, University Hospital Würzburg, Oberdürrbacher Straße 6, Haus A3, D – 97080 Würzburg, Germany

**Keywords:** Transoesophageal echocardiography, Contrast echocardiography, Left atrial mass

## Abstract

Transthoracic and transesophageal echocardiography detected a left atrial mass attached to the intra-atrialseptum. Intravenous contrast agent ruled out atrial thrombus, sugesting a left atrial myxoma. This highlights theimportance of contrast echocardiography for differential diagnosis of left atrial findings.

**Supplementary Information:**

The online version contains supplementary material available at 10.1007/s10554-023-02926-7.

**Figure:**
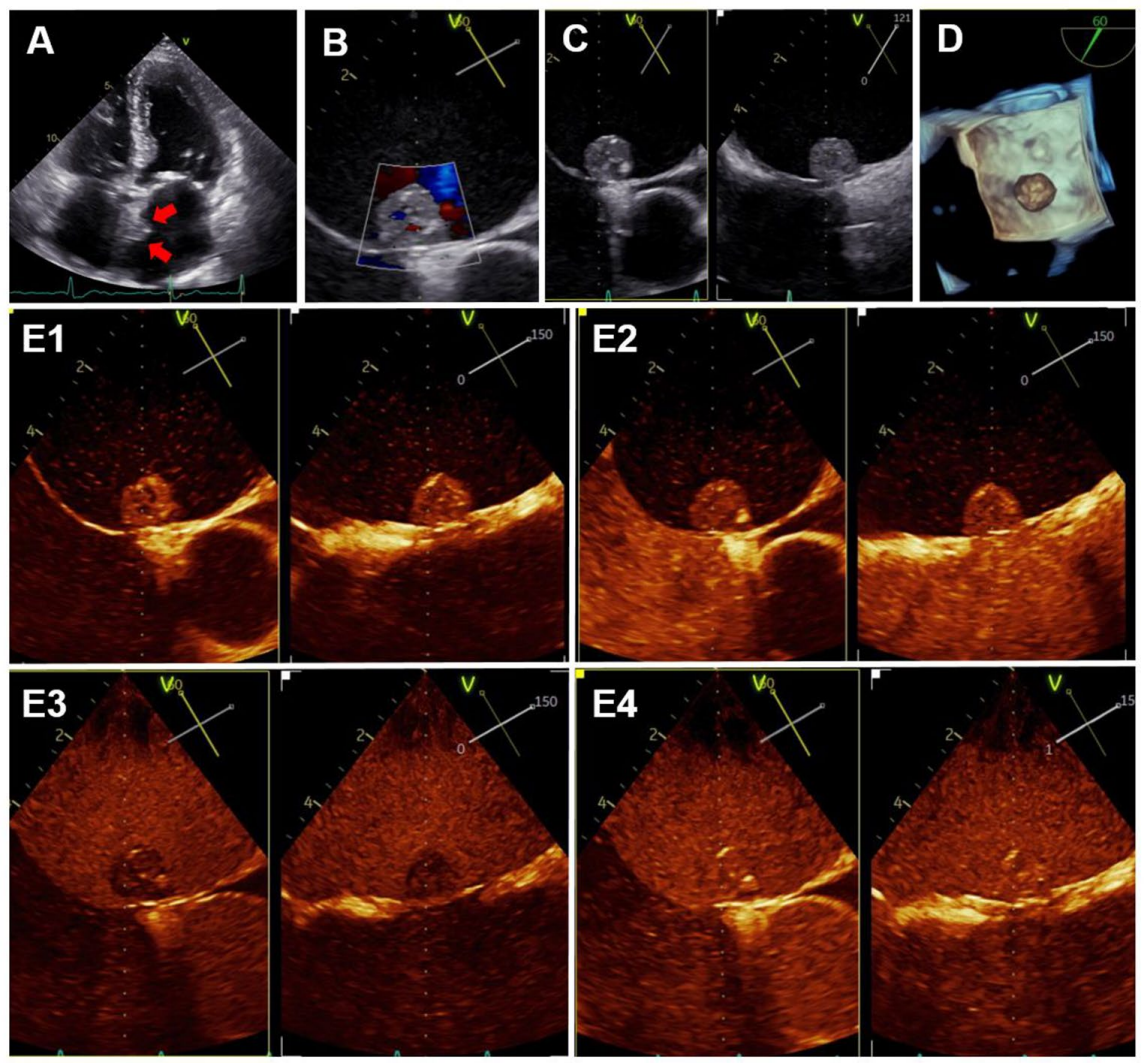

A transthoracic echocardiography in an 83-year-old woman revealed a thickening of the intra-atrial septum/mass in the LA (A). Further diagnostic via transesophageal echocardiography confirmed a 15 × 14 × 14 mm ball-shaped echo-inhomogeneous left-atrial mass originating from the intra-atrial septum without infiltration of adjacent structures (Panels C and D). *Worm-like* anechoic structures beside echo-dense areas and color-Doppler evaluation suggested blood vessels (Panel B). Intravenous SonoVue (Bracco Imaging) echo contrast agent was applied. It showed distribution from the right heart (E2) to the left atrium, initially sparing the intra-atrial septum mass (E3), before it adapted its echogenicity to the left atrial blood pool (E4)(Movie in data supplement).

Application of contrast agent allowed to rule out the differential diagnosis of an atrial thrombus, as only perfused tissue demonstrates uptake of contrast agent. Hence, diagnosis of left atrial myxoma was established. The good vascularization was later also demonstrated by coronary angiography. Due to her age and lack of clinical symptoms, the patient opted against surgery and for a watch-and-wait approach, as informed consent.

This examination should once again exemplify the value of contrast echocardiography - an image modality that in our opinion is too rarely used - as a low-threshold means to exclude relevant differential diagnoses and set the right path for the patient.

### Electronic supplementary material

Below is the link to the electronic supplementary material.

Supplementary Material 1

